# An implantable, intracerebral osmotic pump for convection-enhanced drug delivery in glioblastoma multiforme

**DOI:** 10.3389/fonc.2025.1676691

**Published:** 2025-10-08

**Authors:** Reed Berlet, Azur Azapagic, Neilank K. Jha, Daniil Aksenov, Jade Bookwalter, Ata Ullah, George Bobustuc, John Lee, Himanshu Sant, John McDaid, Matthew Walker, Jill Shea, Dylan Graff, Ann K. Barlow, Roberta Frigerio, Daniel Aliee, Clint Bailes, Bruce K. Gale, Julian E. Bailes

**Affiliations:** ^1^ Department of Neurosurgery, Endeavor Health, Evanston, IL, United States; ^2^ Department of Mechanical Engineering, The University of Utah, Salt Lake City, UT, United States; ^3^ Deep Brain BCI Corp., Wilmington, DE, United States; ^4^ Department of Radiology, Endeavor Health, Evanston, IL, United States; ^5^ Department of Anesthesiology, Endeavor Health, Evanston, IL, United States; ^6^ University of Chicago Pritzker School of Medicine, Chicago, IL, United States; ^7^ Department of Biomedical Engineering, The University of Utah, Salt Lake City, UT, United States; ^8^ Intent Medical Group, Endeavor Health Advanced Neurosciences Institute, Arlington Heights, IL, United States; ^9^ Department of Pathology, Endeavor Health, Evanston, IL, United States; ^10^ Department of Chemical Engineering, The University of Utah, Salt Lake City, UT, United States; ^11^ Department of Surgery, University of Utah School of Medicine, Salt Lake City, UT, United States; ^12^ Centre for Neuroscience Studies, Queen’s University, Kingston, ON, Canada; ^13^ Research Institute, Endeavor Health, Evanston, IL, United States; ^14^ Pritzker School of Medicine, Chicago, IL, United States; ^15^ School of Kinesiology and Health Science, York University, Toronto, ON, Canada; ^16^ College of Engineering, Louisiana State University, Baton Rouge, LA, United States

**Keywords:** glioblastoma, blood-brain barrier, osmotic pump, convection-enhanced delivery, brain implant

## Abstract

**Background:**

Glioblastoma multiforme (GBM; WHO Grade 4) is an aggressive brain tumor that invariably recurs after surgical resection, chemoradiation, and adjuvant chemotherapy. Treatment is limited, in part, because the blood-brain barrier (BBB) restricts entry of chemotherapeutic agents to the brain. Introducing drugs directly into the brain circumvents the BBB, but diffusion of these typically large drug molecules within brain parenchyma is limited. Convection-enhanced delivery (CED), based on the principles of bulk flow, can achieve drug distribution over a wider area to target residual cancer cells and thus remains a promising technique for treating GBM and other neuro-oncologic pathologies. Here, we propose a new method that combines direct brain delivery and CED using a fully implantable, microfluidic pump placed at the time of initial resection surgery.

**Methods:**

In this initial proof-of-concept study, we evaluated the function of a 3D-printed pump in an *in vitro* system and *in vivo* in a rat C6 glioma model.

**Results:**

*In vitro* osmosis-driven distribution of a high molecular-weight marker dye extended up to 18 mm from the pump with minimal reflux, including under simulations of increased intracranial pressure. *In vivo*, MRI imaging demonstrated wide distribution of superparamagnetic iron oxide particles from a pump implanted after the resection of a C6 glioma. Histological staining indicated that pump implantation did not cause additional inflammatory changes compared to controls.

**Conclusion:**

This preliminary study demonstrated the feasibility of using an implantable, osmosis-driven pump to bypass the BBB and provide targeted delivery for treatment of GBM.

## 1 Introduction

Glioblastoma multiforme (GBM) is the most common primary brain tumor, with an age-adjusted incidence rate of 3.2 per 100,000 (1–3). Treatment typically involves maximal safe surgical resection followed by radiation and chemotherapy. Complete surgical removal of the entire tumor is rarely possible and initial recurrence is typically local, in the post-surgical cavity margins; over 80% of GBMs recur within 2 cm of the initial tumor (4–7). Despite the development of new drugs and refinement of radiation techniques, the standard of care chemotherapy for GBM, temozolomide (TMZ), has remained largely unchanged since the advent of chemoradiation in 2005 (8). Overall survival remains poor, with a median survival of 14–15 months and a five-year survival rate of less than 7% (7).

The blood-brain barrier (BBB) is a critical physiological boundary that maintains the homeostasis of the central nervous system (CNS) by regulating the movement of substances between the bloodstream and the brain parenchyma. The BBB is composed of specialized endothelial cells connected by continuous tight junctions, pericytes embedded in the basement membrane, astrocytic end-feet that envelop the vessels, and tight junction proteins that create a physical barrier to paracellular flux. Passage of most macromolecules, pathogens, and hydrophilic compounds into the brain is restricted, while essential nutrients, such as glucose and amino acids, pass through the BBB via specific transport mechanisms (9, 10). Although the local BBB is modified by tumor growth into a blood-tumor barrier, the ability to prevent access of therapeutic agents into the brain is typically unaffected (11).

While essential for protecting neural tissue from toxins and fluctuations in plasma composition, the BBB presents a significant obstacle in treating GBM, as most therapeutic agents, especially large or hydrophilic molecules, cannot access the brain. Thus, drug distribution within the brain is limited to a fraction of the total systemic dose. For example, TMZ levels in cerebrospinal fluid (CSF) were shown to only reach approximately 20% of plasma levels (12). Even small, lipophilic drugs that can cross the BBB may be rapidly removed by active efflux transporters, like P-glycoprotein, that pump substances back into the bloodstream (13, 14). As a result, systemic chemotherapy often fails to achieve therapeutic levels within the tumor site or its zone of recurrence—a limitation that significantly contributes to the poor prognosis in GBM patients (15, 16).

Attempts to bypass the BBB have included several different methods for direct access to the brain. For selected indications, an Ommaya reservoir can be surgically implanted under the scalp to enable repeated delivery of chemotherapeutic agents into the CSF, via ventricular placement, or directly into brain tissue (17). While this device successfully bypasses the BBB to achieve localized drug delivery, spatial distribution of the drug depends on CSF flow and is thus difficult to control, leading to uneven coverage of the target area. Furthermore, implanting and removing such devices requires invasive surgeries, with associated risks of infection and bleeding. Polymer-based drug delivery systems, such as biodegradable Gliadel^®^ wafers, are implanted after tumor resection for local release of carmustine, delivering chemotherapy directly into the resection cavity. However, the polymer degradation rate can be unpredictable, affecting the consistency of drug release. Additionally, the drug diffuses only short distances (2–3 mm) and for a brief duration (24–48 hours), which limits treatment efficacy (18, 19).

Although direct drug delivery approaches can provide localized treatment at the tumor site, drug penetration into the brain relies on diffusion. According to Fick’s Law:


J = −DΔC

where *J* is the flux of molecules through an area, *D* is the diffusivity constant (dependent on molecular size), and Δ*C* is the change in concentration. As diffusion depends on molecular size and concentration gradient, diffusion of large drug molecules is limited to only a few millimeters under typical brain conditions, and thus fails to cover the invasive margins of GBM tumors that extend into the zone of recurrence (20).

Convection-enhanced delivery (CED) is an alternative approach in which a pressure gradient drives drug movement into the brain. CED follows Darcy’s Law:


v = −KΔp

where *v* is the velocity, *K* is the hydraulic conductivity, and Δ*p* is the pressure gradient, which describes the extracellular flow of liquid under a pressure gradient. Under these conditions, drug movement is primarily driven by bulk flow rather than diffusion, reducing the influence of molecular size and concentration gradient on perfusion into the interstitial space. CED enables the rapid distribution of large drug molecules, such as chemotherapeutic agents, over a larger volume of brain tissue than is possible with diffusion alone (21, 22).

The large drug distribution volume achievable with CED may be critical in treating residual local infiltrating tumor within a few centimeters of the original lesion. Because a large concentration gradient is not required for CED, effective local drug distribution can be achieved while significantly reducing systemic toxicity (22, 23). Prior studies have shown that chronic chemotherapy infusion using CED was well-tolerated for up to 10 days (24) and resulted in significantly higher brain concentrations of drug than achieved through systemic (oral or IV) administration (21). Other studies have demonstrated that this approach limits systemic (e.g., liver, bone marrow) toxicity compared to conventional methods (24–46).

The process of CED typically involves placing one or more catheters into brain parenchyma using image (MRI or CT) guidance. The catheters are then attached to an infusion pump that generates pressure, driving the drug solution slowly over a set period of time to target key areas. Although a trial of an internally implanted system is ongoing (39), the infusion pump is usually external and the catheter must be tunneled out of the scalp, so the attendant risk of infection limits treatment duration, and surgeries are required to implant and explant the catheters.

To address these limitations, we evaluated a microfluidic osmotic pump (47) that can be implanted directly into the tumor cavity after resection to deliver targeted therapy directly into the brain. This proof-of-concept study was conducted to evaluate pump performance *in vitro* and in an *in vivo* rat model of GBM. This novel system represents a transformative approach to intracranial drug delivery, combining innovative engineering with fundamental scientific principles to overcome the challenges associated with treating GBM.

## 2 Materials and methods

### 2.1 Microfluidic osmotic pump design

**Figure 1:**
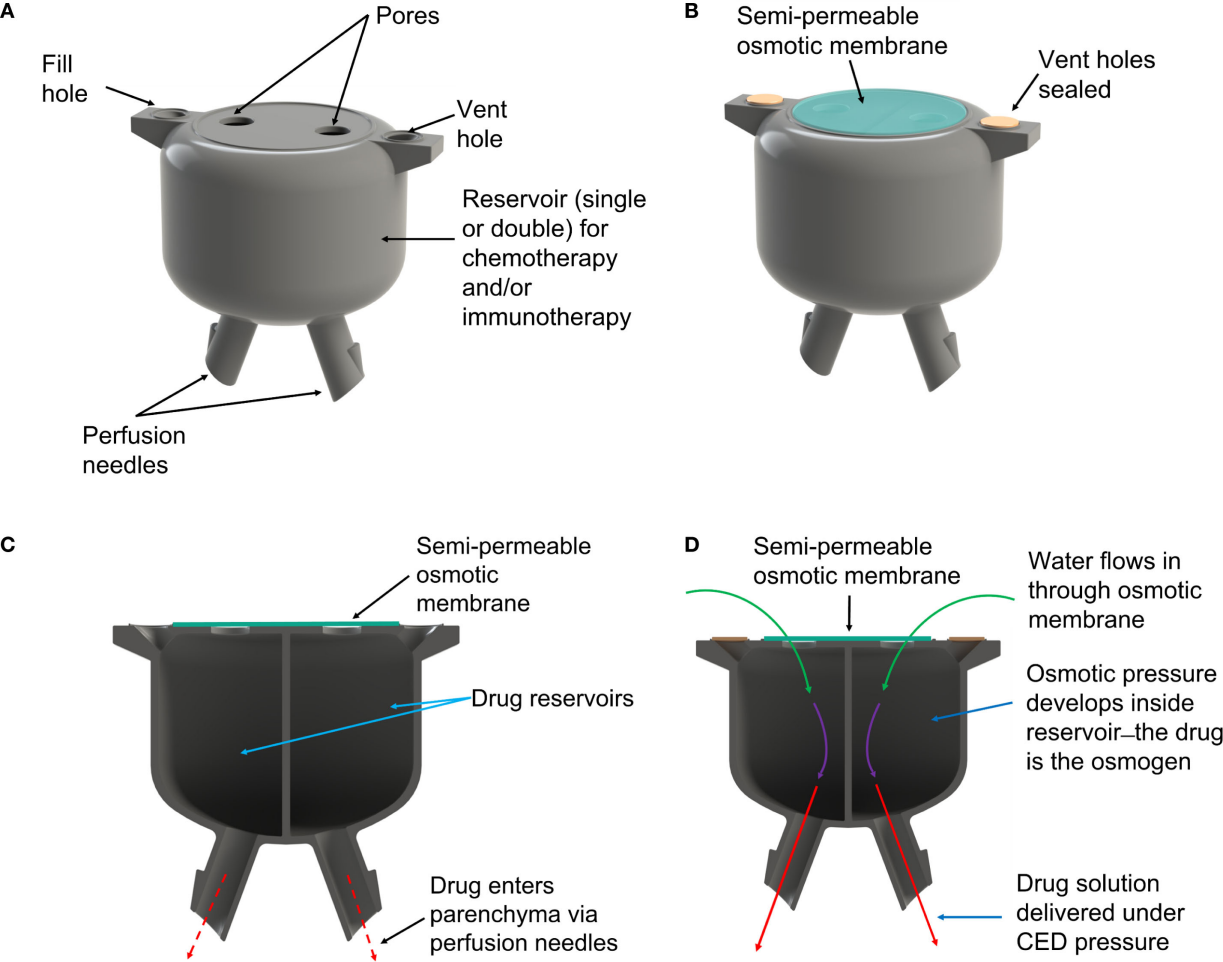
**(A)** Rendering of the 3D-printed osmotic pump. **(B)** After reservoirs are filled with drug solution the vent holes are sealed and an osmotic membrane is placed over the pores. **(C)** The pump comprises two reservoirs allowing use of different drug combinations. **(D)** Water from interstitial fluid is drawn into the reservoirs by osmotic pressure, which forces drug solution out of the perfusion needles and into brain parenchyma.

The osmosis-driven 3D-printed device (**Figure 1**) was designed to achieve controlled drug dosage and targeted delivery through the principles of osmosis-driven flow. The pump was 3D-printed from acrylonitrile butadiene styrene (ABS)-like thermoset MicroFine™ resin on a high-resolution stereolithography printer (Proto Labs Inc.). The device has two independent reservoirs, allowing simultaneous delivery of two distinct therapeutic agents—cytotoxic or immunologic, individually or in combination to enable customized drug combinations and dosages, addressing complex treatment regimens. Pores at the top of each drug reservoir are covered by a one-way osmotic membrane (Millipore™ membrane filters, pore size 25nm), made from hydrophilic, biologically inert mixed cellulose esters (cellulose acetate and cellulose nitrate), which are only permeable to water from the interstitial fluid. The bottom of each reservoir has a small-bore micro-perfusion needle, designed to be placed within the resection cavity walls, directly into the brain parenchyma, up to a depth of 3–5 mm. The micro-perfusion needles are smooth to reduce the risk of trauma to healthy brain tissue.

Once implanted in the resection cavity, the osmotic membrane separates solutions with differing osmolarities: the hyperosmolar drug solution inside the reservoir and the extracellular fluid of the brain. This creates a significant osmotic pressure differential—essentially, the drug solution in each reservoir becomes an osmogen, pulling interstitial water through the osmotic membranes. This ensures that water continuously flows into the reservoirs. According to van’t Hoff’s law:


Π=i·C·R·T

osmotic pressure ( Π ) is proportional to the solute concentration ( C ), temperature ( T ), and the gas constant ( R ); i reflects the degree of ionization of the solute. As water accumulates it generates a hydrostatic pressure ( P ) inside the chamber that drives the drug solution out of the chamber through the perfusion needles, delivering a steady flow of drug precisely to the target site while minimizing drug loss to non-target areas.

### 2.2 *In vitro* testing of the micro-fluidic osmotic pump

The performance of the pump was first evaluated *in vitro* to assess its drug release dynamics and perfusion capabilities. Both reservoirs were filled with a hyperosmolar (25% wt) saline solution containing blue dye (66.6 mg/ml Brilliant Blue FCF) and suspended in a hydrogel (0.2% agarose). The semi-permeable membrane was then exposed to a 0.9% saline solution at room temperature.

The device was run in upright, inverted, and horizontal orientations to investigate whether gravity had any effect on pump performance. Dye release profiles were monitored by taking a photographic image (4K HD Dell camera) every 30 minutes. These images were then optically analyzed using custom image processing software to extract drug delivery rate and perfusion depth data.

The pump was also tested under conditions mimicking increased intracranial pressure, which may occur during tumor growth or after surgery, resulting in reduced drug distribution and/or reflux (backflow of the infused drug along the catheter track). The pump apparatus was subjected to external pressure at 0, 40, 60, and 90 mmHg using a standing water column of the appropriate height, which was attached to the base of a sealed cuvette containing the pump and agarose gel. Pump performance was examined optically at each pressure to check for reflux or pump malfunction, which would be indicated by a lack of dye delivery.

### 2.3 *In vivo* testing of the micro-fluidic osmotic pump

#### 2.3.1 Animals

Adult Sprague Dawley rats (male, 300-450g) were obtained from Charles River Laboratories. All animal procedures were conducted following protocols approved by the Institutional Animal Care and Use Committee (IACUC, study number EH-003) at Endeavor Health Research Institute, ensuring compliance with applicable ethical guidelines. A total of nine animals were used for this study.

#### 2.3.2 C6 glioma cell cultures

Vials containing frozen C6 glioma cells (CCL-107, ATCC) were thawed rapidly with gentle agitation in a 37 °C water bath. Cells were then transferred to a sterile centrifuge tube with 9 mL of complete medium and centrifuged at 125 g for 5 minutes. The supernatant was discarded, and the cell pellet was resuspended in F-12K medium (ATCC) supplemented with penicillin/streptomycin (1%) and fetal bovine serum (2.5%). Cells were cultured in a 25 cm² flask, pre-equilibrated to maintain a pH of 7.0-7.6 at 37°C in a humidified atmosphere with 5% CO_2_ and 95% air.

Cells were subcultured every 2–3 days at a 1:2 to 1:3 ratio. The cell monolayer was washed with Trypsin-EDTA (0.25% (w/v) Trypsin, 0.53 mM EDTA in Hanks Balanced Salt solution without calcium or magnesium, ATCC) to detach cells, which were subsequently reseeded in fresh F-12K medium.

#### 2.3.3 Intracerebral injection of C6 cells glioma cells

C6 glioma cells were prepared for implantation once they reached 60-70% confluence in log growth phase after the second passage. Prior to implantation, cells were washed with 350 µL of phosphate-buffered saline (PBS) and incubated with 200 µL of Trypsin-EDTA. After 5 minutes, cells were centrifuged at 125 g for 5 minutes. The cell pellet was resuspended in Hank’s Balanced Salt solution. The suspension was transferred into a sterile 1 mL syringe attached to a vinyl catheter tube to minimize air bubbles.

Animals were anesthetized using isoflurane inhalation (SomnoSuite, Kent Scientific) at 2-5% for induction and 1-3% for maintenance (with 0.5-1.0 L/min mixed air) and placed in a stereotactic frame under continuous anesthesia. The head was shaved, and the scalp was sterilized with providone-iodine (10%) swab stick (Medline) and 70% isopropyl alcohol (Medline). A midline incision was made to expose the skull, and a 1 mm burr hole was drilled 2 mm anterior and 2 mm lateral to the bregma, using a hand-held drill (KeShi Co. Ltd) and a 1/16 in. drill bit.

A Hamilton 25 µL microsyringe loaded with the cell suspension was used to inject 10 µL (100,000 cells) at a rate of 2 µL/min over 5 minutes. After injection, the burr hole was sealed with bone wax (Ethicon™, Johnson &Johnson), and the incision was closed with simple interrupted sutures. Rats were monitored postoperatively and provided with analgesia (ketoprofen, 4 mg/kg) and supportive care.

#### 2.3.4 Surgical resection

T2-weighted MRI images were acquired using a 9.4 T imaging spectrometer (BioSpec 94/30USR, Bruker Biospin MRI GmbH) to document tumor growth and to confirm tumor size was sufficient to accommodate a pump within resection margins. Rats underwent tumor resection on days 14–20 post-injection.

Resection was performed as described in Bastiancich et al. (48). Briefly, a 5 mm craniectomy was performed around the original injection site, exposing the dura. Tumor mass was removed using a combination of suction and irrigation. Hemostasis was achieved with bipolar cautery (Surgicell Fibrillar) and saline irrigation. The resection cavity was covered with DuraGen (Integra LifeSciences), and the scalp was closed with sutures. Postoperative care included analgesia, antibiotic ointment, and monitoring for deficits.

#### 2.3.5 Pump preparation and implantation

Prior to implantation, pumps were sterilized using the advanced cycle on the STERRAD 100NX hydrogen peroxide gas plasma sterilization system, at temperatures between 45 °C to 55 °C, for 45 to 55 minutes, with a sterilant concentration of approximately 59% hydrogen peroxide. STERRAD-compatible packaging materials were used to ensure optimal sterilization efficacy and material integrity.

In two rats, pumps preloaded with SPIONs (200 μL in 10mL of 10X Tris-buffered saline) were implanted into the resection cavity. Pump reservoirs were filled under sterile conditions using silicone tubing attached to the vent holes, allowing air to escape through the perfusion needles. The vent holes were then sealed using a small amount of bone wax. Pumps were implanted with the perfusion needles aligned along the sagittal plane for optimal MRI imaging of SPION distribution. In four rats, an empty pump was implanted into the resection cavity, and in three rats, no pump was implanted.

#### 2.3.6 MRI imaging of SPION perfusion

Postoperative MRI scans were conducted using the 9.4 T imaging spectrometer and a multi-spin-echo imaging sequence with effective echo time (TE) = 60 ms and Time of Repetition (TR) = 5,000 ms. T2-weighted images were obtained with a field of view of 20 mm × 20 mm and a matrix size of 256 × 256. Imaging was performed on postoperative days 2 and 9 for Rat 1, and days 5 and 8 for Rat 2, to track SPION perfusion.

Perfusion of SPIONS on MRI images were measured using the region of interest (ROI) tool embedded in ParaVision 5.1 software (Bruker) to manually delineate a perfusion area by drawing a boundary defined by the high contrast between SPION-labeled perfusion and surrounding tissue. The boundary was guided by clear signal intensity differences on the MRI images, particularly distinguishing the SPION-enhanced regions. The software computed the ROI area and pixel count to quantify perfusion distribution.

#### 2.3.7 Histology

Tumor tissue was examined histologically to confirm C6 glioma etiology. To determine whether an implanted pump elicited inflammatory responses in brain parenchyma, animals with an implanted pump (no SPIONS, N = 4) or no pump (N = 3) were sacrificed seven days after tumor resection and brains were examined histologically. For euthanasia, anesthesia was performed using isoflurane inhalation at isoflurane 5% (with 0.5-1.0 L/min mixed air) until animals were deeply anesthetized. Euthanasia was by decapitation, using a sharpened guillotine per Endeavor Health IACUC Policy, and brain removal. Pumps were removed, the brains were extracted and subsequently post-fixed overnight in 4% PFA. Coronal brain slices (5–10 µm) encompassing the resected tumor site/pump placement area and tumor slices were prepared using a standard paraffin-embedding procedure. Slices were stained with hematoxylin and eosin (H&E) to assess overall tumor histological architecture, parenchymal infiltration, and/or necrosis. All stained sections were independently reviewed by a board-certified neuropathologist who was blinded to the treatment assignments. The extent of inflammatory cells, surrounding gliosis, and any other pathological findings were recorded.

### 2.4 Results

#### 2.4.1 *In vitro* testing

**Figure 2:**
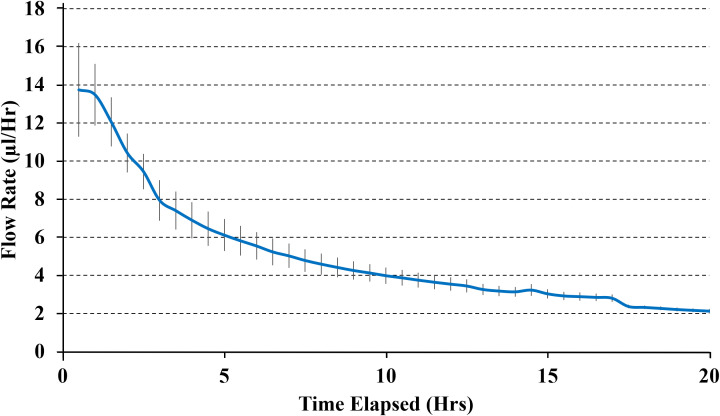
The average delivery rate of pumps with 3mm needles, 25%wt internal salt concentration, 100 μm diameter semi-permeable membrane with 25 nm pores. Results show the average delivery rate from three pumps.

**Figure 3:**
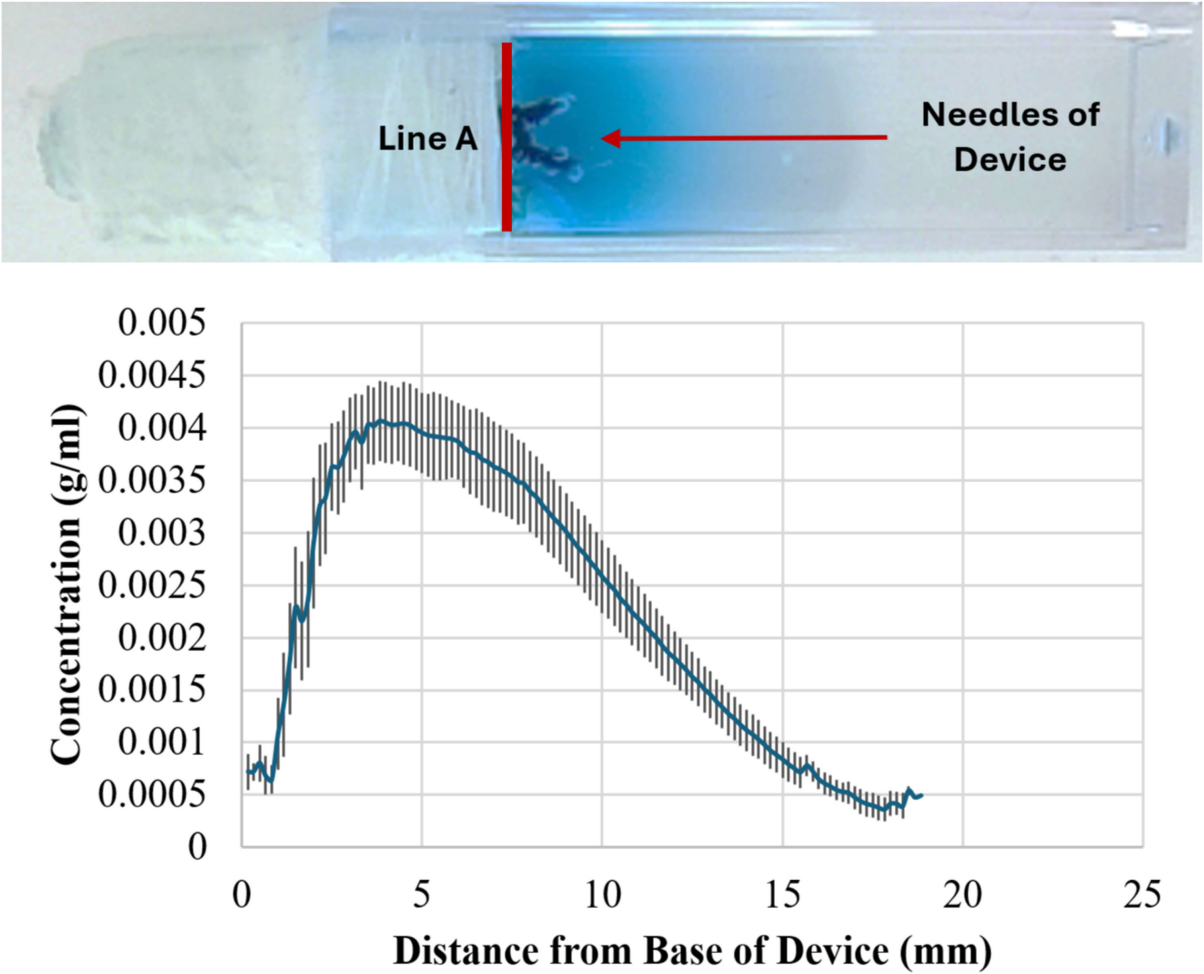
Measuring perfusion distance of dye from osmotic pumps. Top: Representative image of perfusion after 20 hrs. Bottom: Graph showing averaged perfusion of dye from six pumps. The dye perfused ~ 18 mm from the base of the device (Line A in the photograph) which corresponds to 0 mm on the x-axis.

In all experiments, the pumps demonstrated a stable release rate driven by osmotic pressure. No dye was released in the absence of osmotic pressure. The system achieved sustained release consistent with theoretical, *in vitro* engineering predictions in all orientations and combination of different needle lengths, osmogen concentrations, membrane pore sizes, and membrane diameters, ensuring steady therapeutic levels without abrupt fluctuations. A representative result is shown in **Figure 2**. Furthermore, the pump delivered drugs effectively into the surrounding medium, achieving a penetration depth of up to 18 mm from the edge of the pump (Line A), which corresponds to the edge of the resection cavity. Results from six pumps were averaged to determine an overall release profile (**Figure 3**).

The mechanical integrity of the reservoirs was maintained under simulated physiological conditions, with no leakage or structural failure observed. Even when placed under external pressure up to 90 mmHg, the system continued functioning as intended, maintaining sustained drug delivery with minimal reflux.

*In vitro* testing suggested that the pump would deliver the hyperosmotic drug mixture stored within the reservoir under expected physiological conditions, with minimal reflux, when implanted in the brain.

#### 2.4.2 *In vivo* testing

##### 2.4.2.1 SPION distribution from implanted pump

**Figure 4:**
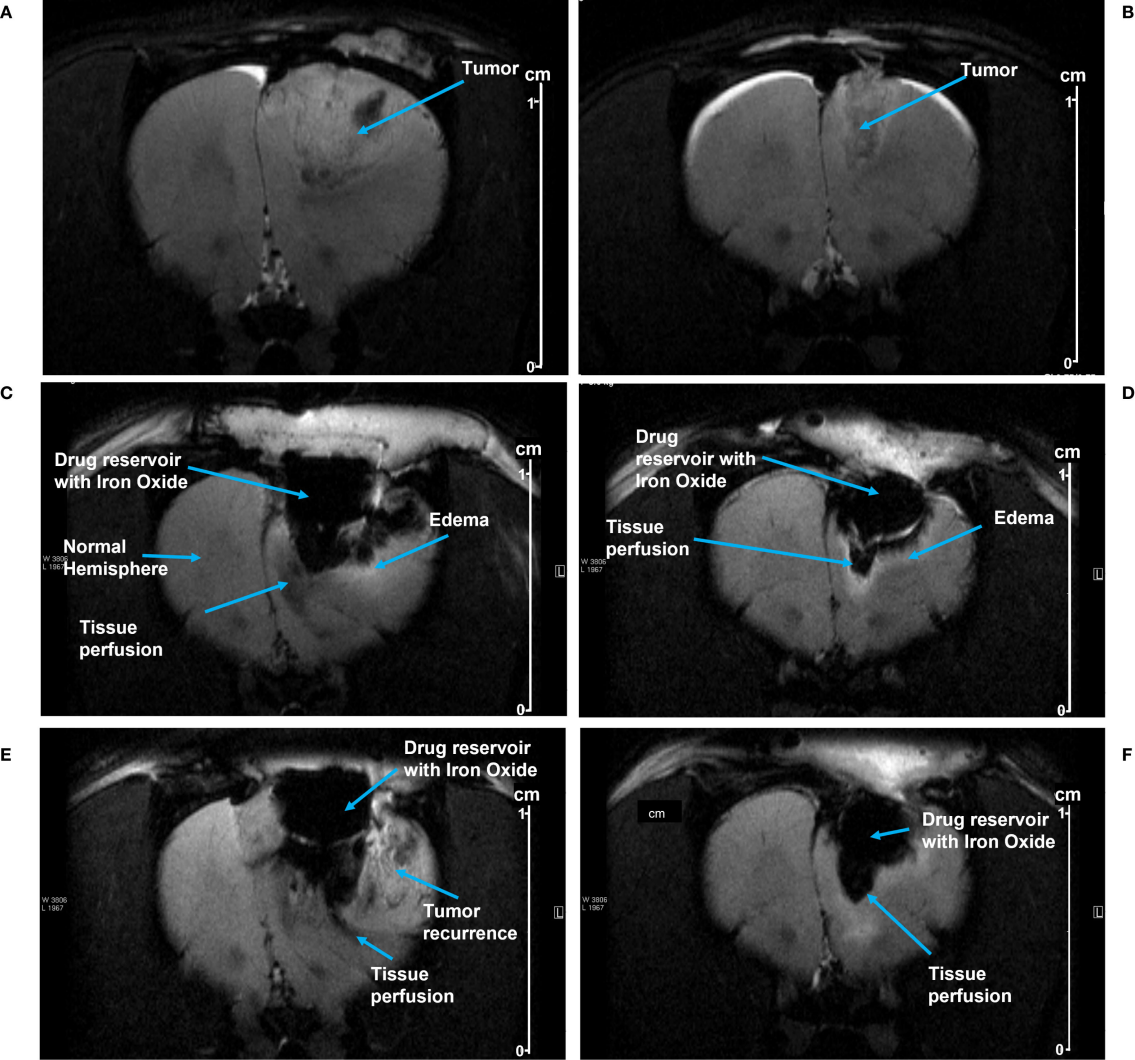
**(A, B)** show MRI images of tumor growth prior to resection in Rat 1 and Rat 2, respectively. After pump implantation, MRI images show SPION perfusion over time into the tumor resection cavities in Rat 1 at day 5 **(C)** and at day 8 **(E)**; and in Rat 2 at day 2 **(D)** and at day 9 **(F)**. The perfusion area was manually delineated beneath the pump, using the distinct contrast between iron oxide and the surrounding tissue (see the methods). Scale shown at right (cm).

A rat C6 glioma model was used to evaluate pump function *in vivo*. Growth of the tumor was monitored by MRI. **Figures 4A, B** depict tumor growth in two animals, Rat 1 and 2, respectively; Rat 1 was imaged on Day 12 post-injection, and Rat 2 was imaged on Day 13 post-injection. Tumors were resected from both rats and pumps implanted on Day 14 post-injection; two weeks of tumor growth provided a resection cavity of sufficient size to implant a pump.

MRI images from Rat 1 show pump activity on days 5 and 8 post-implantation (**Figures 4C, E** respectively). Fluid/SPION is evident in the inferior portion of the pump in both C and E, along with subtle edema. A hyperintense T2 signal was observed in the parenchyma inferior and lateral to the pump chamber, indicating edema and/or fluid related to inflammation and/or tumor recurrence. Beneath the pump in the tissue perfusion zone, T2 hypointensity was noted, representing SPION perfusion and correlating with the signal intensity findings shown in **Table 1**. **Figures 4D, F** show MRI images from Rat 2 on post-implantation days 2 and 9, respectively. Again, SPIONs were observed in the inferior and lateral portions of the pump. However, in this rat there was no indication of tumor recurrence and minimal inflammation. A subtle hyperintense T2 signal along the tumor fluid interface is likely attributable to edema/inflammation. Beneath the pump, the T2 hypointensity correlated with SPION perfusion.

**Table 1:** SPION perfusion over time.

Animal	Days post resection/pump implantation	Area of perfusion (cm^2^)
1	5	0.01
	8	0.03
2	2	0.04
	9	0.10

**Figure 5:**
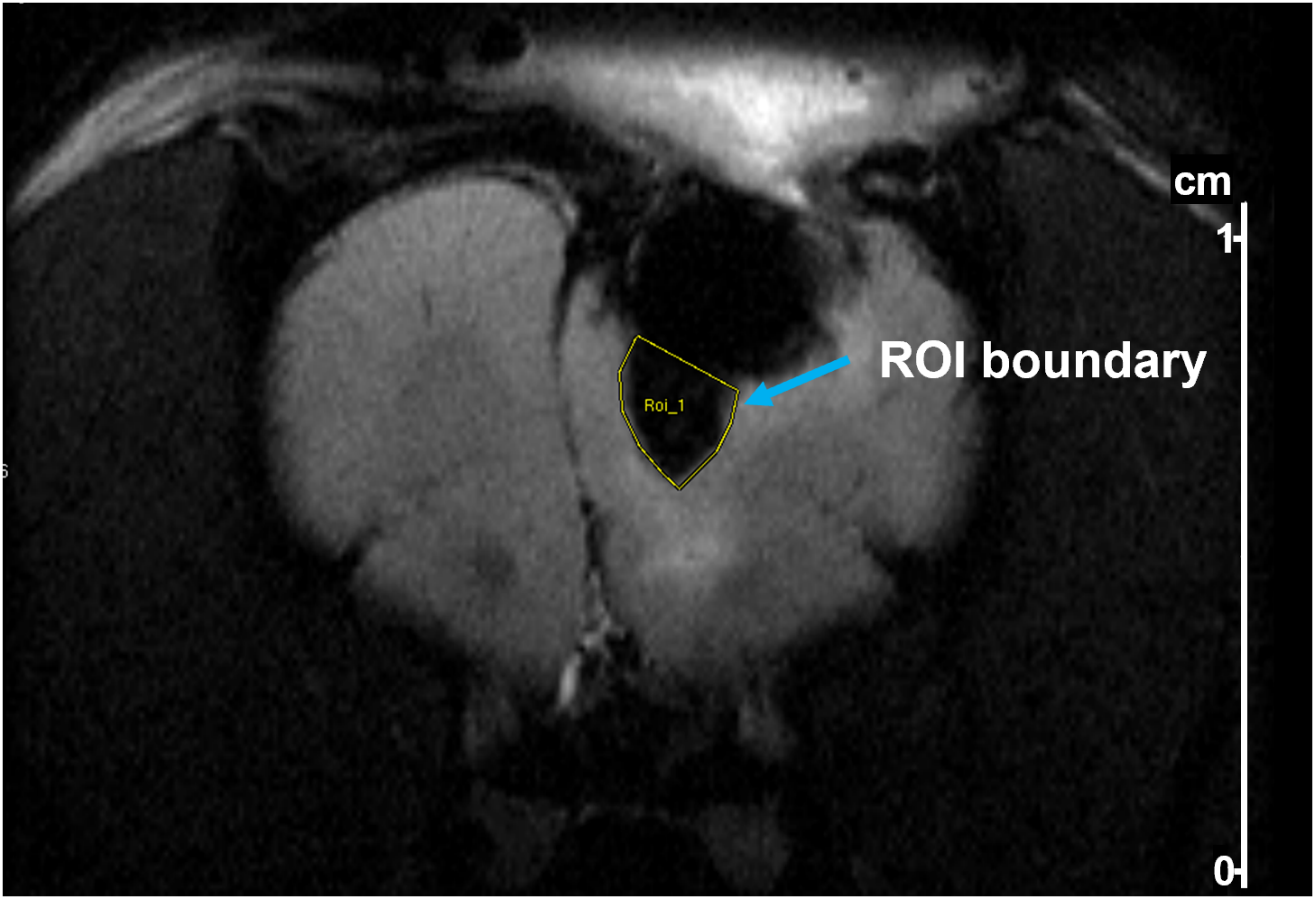
Example of delineation of a region of interest (ROI) in a T2-weighted MR image of a rat after tumor resection and pump implantation. The ROI (yellow outline) was drawn to quantify SPION perfusion below the pump. Delineation was performed using Bruker ParaVision software based on contrast between iron oxide-labeled regions and surrounding brain tissue.

SPION perfusion was calculated from MRI images using the ParaVision software tool to draw a polygon boundary defined by the high contrast between SPION-labeled perfusion and surrounding tissue (an example is shown in **Figure 5**). For Rat 1, the area of SPION perfusion increased by 0.02 cm² over 3 days, while for Rat 2, it increased by 0.06 cm² over 7 days (**Table 1**).

##### 2.4.2.2 Histology

**Figure 6:**
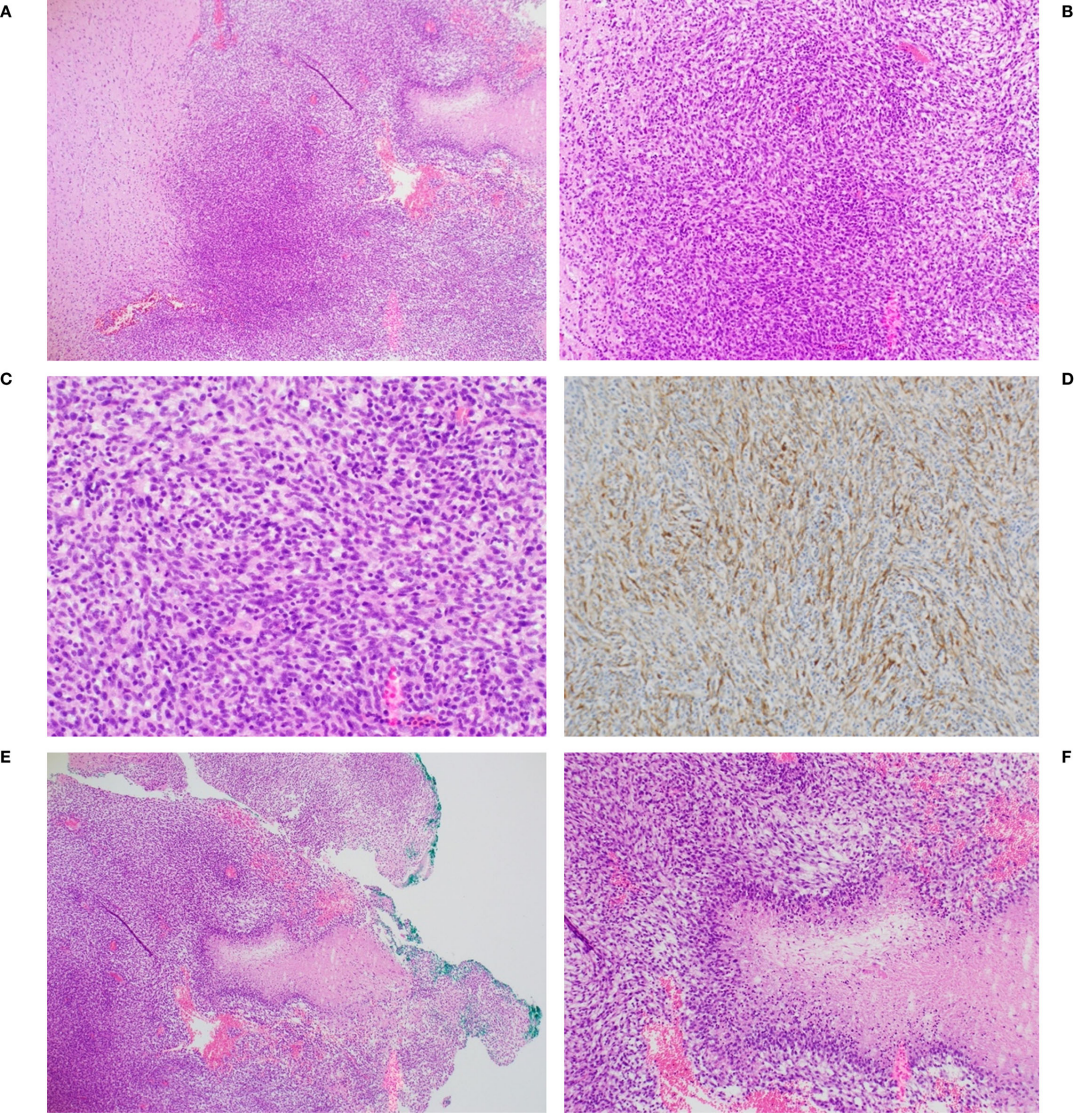
Representative histopathology of C6 glioma from one animal. Confluent tumor seen at **(A)** 4X, **(B)** 10X and **(C)** 20X magnification. **(D)** tumor was positive for glial fibrillary acidic protein (GFAP), 10X magnification. Palisading necrosis architecture, characteristic of a high grade glioma (GBM) [also seen to the right in panel **(A)**] is shown at 4X **(E)** and 10X **(F)** magnification.

**Figure 6** illustrates characteristics of a representative C6 tumor that confirmed its designation as a Grade 4 glioma. Panels A, B and C show a confluent and poorly differentiated tumor stained with H&E with signs of neovascularization. Panel D is the same tumor stained with antibodies to glial fibrillary acidic protein (GFAP), indicating that it was non-sarcomatous and of glial origin. Panels E and F show the palisading necrosis typically seen in Grade 4 gliomas.

**Figure 7:**
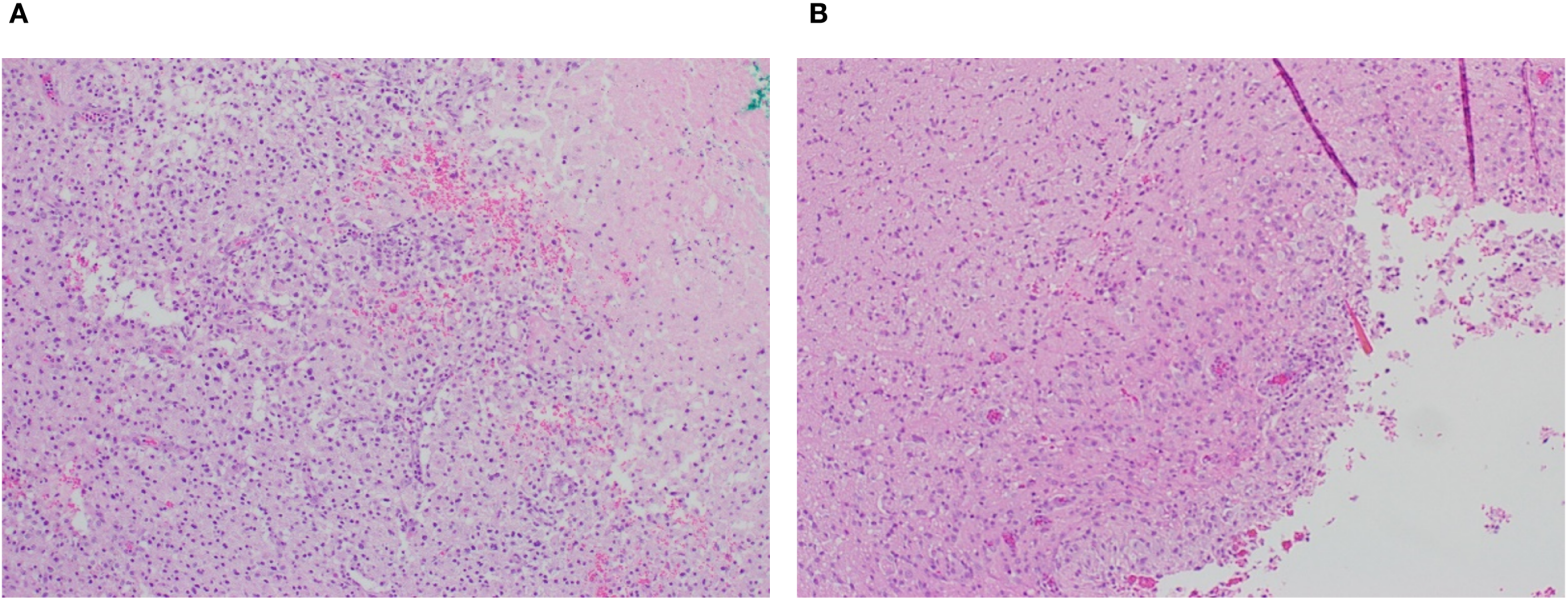
Representative post-resection tumor cavity **(A)** without and **(B)** with pump placement (4X magnification). Presence of macrophages and chronic inflammation is similar under both conditions.

Histological examination seven days after tumor resection in animals with or without pump implantation showed a well-defined resection cavity at the site of tumor removal. No gross abnormalities, such as hemorrhage or excessive necrosis, were observed in either group beyond that typically associated with a surgical resection (**Figure 7**). H&E staining showed inflammatory infiltrates, including lymphocytes and activated macrophages, in both groups. In addition, no substantial differences between groups in perilesional edema; extent of focal residual tumor; or tissue reaction, including macrophages, chronic inflammation or necrosis, were noted.

## 3 Discussion

*In vitro* testing of the osmosis-driven 3D-printed microfluidic osmotic pump in 0.2% agarose gels demonstrated robust functionality and the ability to achieve sustained and controlled drug release driven by osmotic gradients, with effective distribution reaching up to 18 mm, far exceeding the limitations of diffusion-based direct delivery systems. The system successfully minimized drug reflux under simulated conditions mimicking increased intracerebral pressure, as may occur due to brain swelling in response to a tumor or after resection.

*In vivo* studies confirmed these findings, showing SPION perfusion into brain tissue, with the distribution of SPIONs increasing over time. The results from this proof-of-concept study collectively highlight the reliability and versatility of the implanted device, providing a solid foundation for its continued development toward clinical applications in neurological conditions such as GBM. Further optimization and expanded testing are required to enhance the translational potential of the system and readiness for clinical use.

Histological assessment indicated that implantation of the osmotic-driven CED pump after C6 glioma resection did not cause increased local inflammatory responses in rat brain tissue compared to controls with no pump implanted, seven days after implantation. Although surgical manipulation itself can induce gliosis and immune cell infiltration, our results suggest that the osmotic pump hardware did not result in increased or prolonged post-surgical inflammation; longer term implantation of a microchip in rat brain similarly resulted in minimal inflammation (49). However, further studies with larger cohorts and longer observation periods are necessary to fully characterize any delayed inflammatory processes associated with device implantation.

This novel design overcomes multiple barriers to the implementation of CED, including the need for a catheter protruding from the scalp and an external drug pump, while offering enhanced direct targeting of parenchyma away from pial or ventricular surfaces, eloquent areas, and other unexpected findings during tumor resection (6, 32, 33, 37, 50). Placing the micro-perfusion needles directly into the brain tissue is intended to bypass any irregularities of the resection cavity, such as regions of necrosis, fibrotic areas, or cautery artifacts that may otherwise affect drug distribution. The use of perfusion needles and hydrostatic pressure-driven flow prevents the backflow of the drug solution along the perfusion pathway, ensuring that the therapeutic agent remains confined to the intended delivery region and reducing the risk of off-target toxicity. By modulating the osmolarity of the drug solution, the rate of water influx and, consequently, drug dosage delivered over time, can be adjusted. Together, the innovative features of this implantable pump enable controlled, targeted drug delivery, reducing systemic side effects and potentially improving therapeutic outcomes.

### 3.1 Study limitations

While this study demonstrated the feasibility of using a microfluidic pump for direct intracerebral drug delivery, this was an initial proof-of-concept study and thus has some limitations.

The small sample sizes preclude generalization; further studies with larger sample sizes are required to confirm these findings. While the study indicated that pump implantation did not cause local inflammation during the study timeframe, the long-term effects of pump implantation within the brain were not evaluated. Long-term studies are needed to evaluate the effects on brain tissue, as well as on global neurological and survival outcomes for disease states. While *in vitro* studies showed that the pump functioned under various simulated intracranial pressures and under physiological pressure *in vivo*, our study did not evaluate pump function under higher pressures or under conditions of fluctuating intracranial pressure that may occur, for example, after surgery or during tumor regrowth.

The implanted pumps in this study were not used to deliver drugs, thus therapeutic effects could not be evaluated. Future studies are required to examine the effects of intracerebral drug delivery on tumor growth. Although the C6 glioma cell line is a commonly used glioma model, a single cell line may not fully represent the cellular and molecular heterogeneity or growth characteristics of human GBM, thus other cell lines or animal models may be required to fully evaluate efficacy.

### 3.2 Future directions

For clinical applications, the pump can be fabricated using FDA-approved biodegradable polymers, such as polylactic acid (PLA) or polycaprolactone (PCL), that degrade into non-toxic byproducts (e.g., lactic acid), which are metabolized and excreted by the body. The use of biodegradable materials would allow the device to maintain functionality over a defined therapeutic period, after which it would safely degrade, eliminating the need for surgical removal. These materials are mechanically stable, ensuring durability during the implantation period, and their degradation rate could be controlled to align with the therapeutic timeline. A controlled lifespan would ensure that drug delivery is terminated once therapy is complete, preventing overdosing or unnecessary prolonged exposure. Further refinements of pump design to combine delivery of sustained, prolonged (6 weeks), cytotoxic drug concentrations with delayed initiation of delivery (~10 days) and a predetermined life span (~10–12 weeks to ensure completion of full treatment course) before pump resorption may allow placement of a drug-loaded microfluidic osmotic pump at the time of initial GBM resection to replace the 6-week oral TMZ treatment during upfront chemoradiation.

Incorporating intraoperative imaging techniques, such as real-time MRI, fluoroscopy or ultrasound guidance, or leveraging neural mapping data from techniques like functional MRI or electroencephalography (EEG) could inform the precise placement of the pump in functionally critical regions, such as motor or speech centers, thereby optimizing therapeutic outcomes while preserving vital brain functions. Accurate localization would allow the device to deliver drugs directly to specific brain regions, such as a tumor margin or a damaged cortical area, ensuring higher efficacy. Improving the targeting capabilities of the osmosis-driven pump may involve integrating advances in imaging, device customization, biosensing, and biochemical monitoring.

Tailoring the device’s shape, reservoir configuration, and needle length to specific brain regions could improve targeting in anatomically critical areas. Multiple perfusion needles with adjustable lengths and orientations could be used to improve drug distribution across complex tissue geometries. This approach would be particularly beneficial for treating irregularly shaped tumors or multifocal disease sites. Adapting the perfusion needles for stereotactic guidance could enhance their ability to reach deep or hard-to-access areas with millimeter-level accuracy. Combining this with robotic assistance for pump placement could further minimize the risk of collateral damage to healthy tissues. These enhancements could further refine drug delivery to specific regions or tissue types, enabling treatment of diverse neurological conditions in addition to GBM, including epilepsy and neurodegenerative diseases. By aligning drug delivery with the brain’s complex anatomical and functional architecture, these improvements would maximize therapeutic efficacy while minimizing off-target binding and potential damage to normal tissues.

Future large animal studies are needed to further evaluate pump delivery dynamics of drug or drug combinations, risk of local toxicity, infection risk, immune or inflammatory responses to the implant and/or degradation products, and how the implanted device affects surrounding healthy brain tissue. Use of pumps made from biodegradable materials that would not require explantation is an important feature of the proposed pump system, and such materials are widely used in neural tissues; however, the viability and long-term safety of these materials in the brain must be evaluated. Contingency measures in the event of pump malfunction or blockage must be considered. Methods to monitor drug distribution and studies to develop treatment protocols, drug combinations, and infusion rates must also be determined to optimize intracerebral treatment of GBM. While outside the scope of this proof-of-concept study, these questions will direct next steps towards clinical translation of this novel device.

## Data Availability

The original contributions presented in the study are included in the article/Supplementary Material. Further inquiries can be directed to the corresponding author.
